# Transcriptomics unravels molecular changes associated with cilia and COVID-19 in chronic rhinosinusitis with nasal polyps

**DOI:** 10.1038/s41598-023-32944-3

**Published:** 2023-04-21

**Authors:** Åsa Torinsson Naluai, Malin Östensson, Philippa C. Fowler, Sanna Abrahamsson, Björn Andersson, Stina Lassesson, Frida Jacobsson, Martin Oscarsson, Anton Bohman, Ali M. Harandi, Mats Bende

**Affiliations:** 1 Department of Laboratory Medicine, Institute of Biomedicine, Sahlgrenska Academy at the University of Gothenburg, Gothenburg, Sweden; 2 Core Facilities, Sahlgrenska Academy at the University of Gothenburg, Gothenburg, Sweden; 3 Department of Otorhinolaryngology, Skaraborg Hospital, Skövde, Sweden; 4 Department of Otorhinolaryngology, Uppsala University Hospital, Uppsala, Sweden; 5 Department of Microbiology and Immunology, Institute of Biomedicine, Sahlgrenska Academy at the University of Gothenburg, Gothenburg, Sweden; 6 Vaccine Evaluation Center, BC Children’s Hospital Research Institute, University of British Columbia, Vancouver, Canada

**Keywords:** Biochemistry, Biological techniques, Cell biology, Genetics, Biomarkers, Diseases, Molecular medicine

## Abstract

Chronic rhinosinusitis with nasal polyps (CRSwNP) is a common upper respiratory tract complication where the pathogenesis is largely unknown. Herein, we investigated the transcriptome profile in nasal mucosa biopsies of CRSwNP patients and healthy individuals. We further integrated the transcriptomics data with genes located in chromosomal regions containing genome-wide significant gene variants for COVID-19. Among the most significantly upregulated genes in polyp mucosa were *CCL18*, *CLEC4G*, *CCL13* and *SLC9A3*. Pathways involving “Ciliated epithelial cells” were the most differentially expressed molecular pathways when polyp mucosa and non-polyp mucosa from the same patient was compared. Natural killer T-cell (NKT) and viral pathways were the most statistically significant pathways in the mucosa of CRSwNP patients compared with those of healthy control individuals. Upregulated genes in polyp mucosa, located within the genome-wide associated regions of COVID-19, included *LZTFL1*, *CCR9*, *SLC6A20*, *IFNAR1*, *IFNAR2* and *IL10RB*. Interestingly, the second most over-expressed gene in our study, *CLEC4G*, has been shown to bind directly to SARS-CoV-2 spike's N-terminal domain and mediate its entry and infection. Our results on altered expression of genes related to cilia and viruses point to the de-regulation of viral defenses in CRSwNP patients, and may give clues to future intervention strategies.

## Introduction

The human nasal mucosa is the main portal of entry and a critical site of infection of respiratory pathogens. Chronic rhinosinusitis with nasal polyps (CRSwNP) is a common upper respiratory tract complication in humans^1^. CRSwNP is a condition defined by chronic inflammation of the nasal cavity and the paranasal sinuses combined with bilateral polyps in the middle meatus of the nose. CRSwNP is difficult to treat, and recurrences are frequent, despite medical treatment and surgical interventions. The clinical management of CRSwNP is largely ineffective partly due to the limited understanding of the underlying pathogenic factors. Therefore, understanding the mechanisms that underlie CRSwNP pathogenesis is essential to help identify targets and/or pathways for therapeutic interventions.

Despite several hypotheses over the years, the pathogenesis of CRSwNP remains unclear^2^. Studies have shown an increased frequency of positive family history among those affected^1,3,4^ and there is a five-fold increased risk of having CRSwNP among first-degree relatives to subjects with the disease, indicating that genetic mechanisms are important^1^.

Studies of gene expression in nasal polyps have shown significantly altered expression levels of several genes when comparing nasal polyp tissue to nasal mucosa from unaffected individuals^5–11^. A previous study based on gene expression microarray of six nasal polyp samples from patients with CRSwNP and six tissue samples from control subjects, suggested induction of type 2 inflammation and eosinophil migration, such as *CCL13*, *CCL18*, *CCL8* and genes related to antimicrobial defense responses^12^. Similarly, using microarray technology, whole gene expression has been studied in several chronic respiratory sinusitis (CRS) populations^13–16^.

A few previous whole transcriptome RNA sequencing analyses of CRSwNP tissue have also been performed^17–19^. A report from Wang et al. examined specifically patients with CRSwNP and comorbid asthma, where they analysed ten patients with CRSwNP and asthma, ten patients with CRSwNP-alone and nine healthy control individuals. This study identified the HISLA Gene “HIF1A Stabilizing Long Noncoding RNA” (alias LINC01146) as a hub lncRNA dysregulated in CRSwNP patients, with and without asthma, compared with controls^17^.

Another RNA sequencing study collected mucosal tissue samples from six CRS without nasal polyps (CRSsNP), six CRSwNP, and six control patients. Additional matched polyp samples were also collected from the six CRSwNP patients. In their study, CRSwNP polyp tissue showed an upregulation of B-cell mediated immune responses and a reduced expression of genes related to epithelial morphogenesis and homeostasis. Finally, the largest transcriptome analysis of CRSwNP so far has been performed by Peng et al.^19^ who performed RNA sequencing analysis on 44 CRSwNP patients and 41 control subjects. This study reported the transcript signatures of CRSwNP as genes involved in cilia dysfunction, interferon signaling, viral responses, inflammation and abnormal metabolism of extracellular matrix (ECM)^19^.

Despite the growing body of reports on differentially expressed genes and pathways associated with CRSwNP, the complex disease mechanism and pathways underlying CRSwNP remain elusive and require further investigation. Given that, the aim of this study was to add to the knowledge of the transcriptome profile in CRSwNP using 50 nasal mucosa biopsies, integrate genome wide genetic risk variants and specifically investigate genes located within genome-wide significant regions in COVID-19.

## Materials and methods

### Ethical statement

The study was done in accordance with the principles expressed in the Declaration of Helsinki and was approved by the Regional Ethics Committee in Gothenburg, Sweden. A written informed consent was obtained from all participants in the study. The confidentiality of the personal data from all participants was ensured throughout the study.

### Biological materials

Polyp tissue was collected from 16 CRSwNP patients with a mean age of 60 years (range 35–73) and a total of ten males and six females. Two types of control tissues were used: adjacent non-polyp tissue from inferior/middle turbinates of the 16 CRSwNP patients, and inferior/middle turbinates from 18 healthy controls with no history of sinus disease with a mean age of 59.8 years (range 40–84) and a total of eight males and ten females. The patient and control populations show a somewhat uneven distribution for gender, with 38% females in CRSwNP patients and 63% females in the controls. For females the age ranged from 38 to 76 years old with a median age of 63. Male age ranged from 35 to 84 with a median of 57. Control patient age ranged from 40 to 84 years old with a median age of 60 and CRSwNP patient age ranged from 35 to 73 with a median of 60.

All biopsies were immediately put in RNA later preservative fluid (ThermoFisher, San Diego, CA, USA). The diagnosis of CRS was based on the definition of the European Position Paper on Rhinosinusitis and Nasal Polyps guidelines^20^. All CRSwNP patients were treated locally in their nasal cavity with corticosteroids as recommended by the current Rhinosinusitis and Nasal Polyps 2012 guidelines^20^. The study was carried out in accordance with the Declaration of Helsinki and was approved by the Local Ethics Committee in Gothenburg, Sweden (Date for approval 2012-12-30, Dnr 829-12).

### RNA extraction and cDNA synthesis

The 50 tissue samples from CRSwNP patients and healthy controls were directly put in RNA later medium (Thermo Fisher Scientific Inc., CA, USA) and processed for extraction and purification of total RNA using the Kingfisher RNA kit together with the Kingfisher instrument (Thermo Fisher Scientific Inc., CA, USA) according to the manufacturer’s instructions. The quantity and quality of isolated RNA was determined using the NanoDrop 2000 Spectrophotometer (Thermo Fischer Scientific) and 2200 TapeStation Automated Electrophoresis System (Agilent Technologies). Samples with an RNA integrity number of greater than 6.0 were chosen for sequencing, with the exception of one polyp sample at RIN 4 which was included.

### Whole genome RNA sequencing

Preparation of libraries was carried out using the TruSeq Stranded Total RNA Sample Preparation Kit with Ribo-Zero Gold (Illumina, Inc., San Diego, CA), using 60–1000 ng of total RNA input. The Novaseq 6000 platform was used (Illumina, Inc., San Diego, CA) and 100 bp paired-end reads were generated by Clinical Genomics (Gothenburg, Sweden).

Adapters and low-quality tail were trimmed from reads prior to read alignment. Clean sequence reads aligned to the human genome were used to assemble transcripts, estimate the abundance of these transcripts and detect differential expression among samples. For mRNA and lncRNA analyses, the reference genome build GRCh38/hg38 was chosen as the annotation references. Fragments per kilo-base of exon per million fragments mapped (FPKM) of both lncRNAs and mRNAs in each sample were calculated based on the length of the fragments and reads count mapped to this fragment.

### Data availability statement

Raw sequence reads in FASTQ format were evaluated in terms of read quality (per base sequence quality, per base G + C content, sequence length distribution, sequence duplication levels, Kmer content and low complexity sequences). The quality statistics were gathered using FastQC^21^ (version 0.11.2) available at https://www.bioinformatics.babraham.ac.uk/projects/fastqc/.The resulting reports were merged using MultiQC^22^ version 0.9. Quality filtering of reads and adapter removal was performed using Trim Galore^23^ (0.4.0) together with Cutadapt (1.9)^24^.

All data is available on the European Genome-phenome Archive ID for data set: EGAD00001010146, ID for study: GAS00001007088, or by contacting the corresponding author.

### Mapping and read quantification

The quality filtered reads were mapped with STAR (2.5.2b) towards the human reference genome (GRCh38).^25^ Read quantification was performed with featureCounts (1.6.4).^26^.

### Principal component analysis

Principal component analysis (PCA) was carried out in R version 4.1.3 (www.r-project.org)^27,28^ using log2 reads per kilobase million (RPKM) to determine the differences between the samples in each group. Using the module, the principal components of the three tissue types CRSwNP-polyp, CRSwNP-adjacent non-polyp tissue and healthy control tissue, were estimated.

### Differential expression analysis

For differential gene expression discovery, the DESeq2 R package was used^29^. A variance stabilizing transformation (vst) was applied to gene expression counts. To identify differentially expressed genes (DEGs), an adjusted *p*-value < 0.01 and 0.001 were set as thresholds to define the significance in the three comparisons: Control vs non polyp mucosa from CRSwNP patient, non-polyp CRSwNP patient mucosa vs polyp mucosa from the same patient with CRSwNP and finally control vs polyp mucosa from CRSwNP patient. Due to an uneven matching on age and gender between the CRSwNP patient and controls, gender and age were used as covariates in all the statistical analyses using DESeq2. Heatmaps were generated using “pheatmap” in R, R version 4.1.3.

### Pathway enrichment analysis

Lists of the most differentially expressed genes (DEGs) in each comparison were imported into GeneTrail 3.0^30^ (https://genetrail.bioinf.uni-sb.de/start.html) for pathway enrichment analysis, and the top significantly enriched KEGG^31^, Wiki, Panther and Reactome pathways as well as tissue type/cell marker overrepresentation, provided by GeneTrail 3.0, were determined.

## Results

### Profiling DEGs in the polyp mucosa of CRSwNP patients and adjacent non-polyp mucosa from the same CRSwNP patient

**Figure 1:**
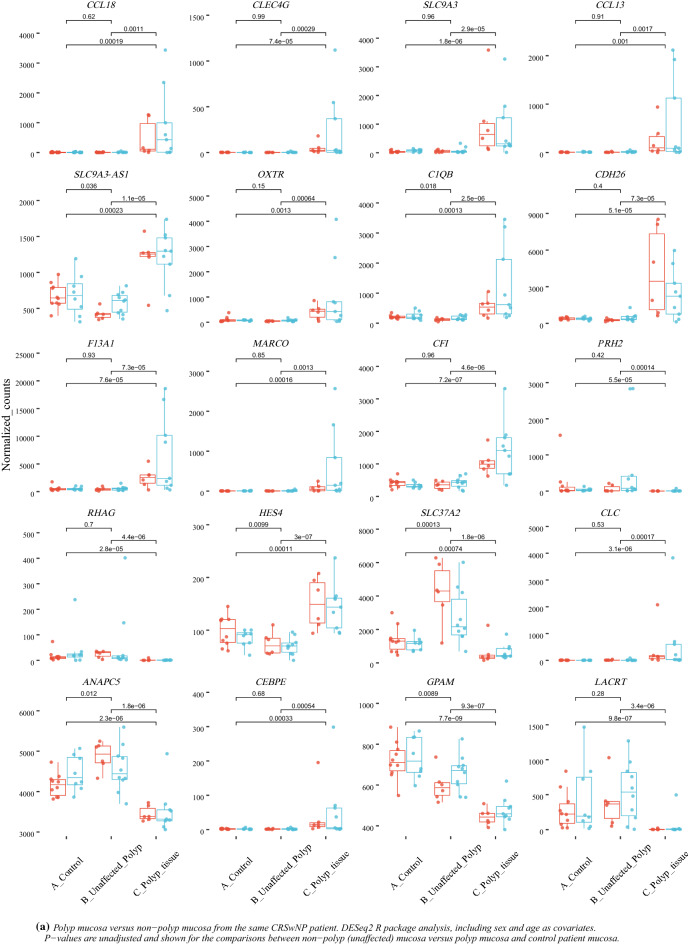

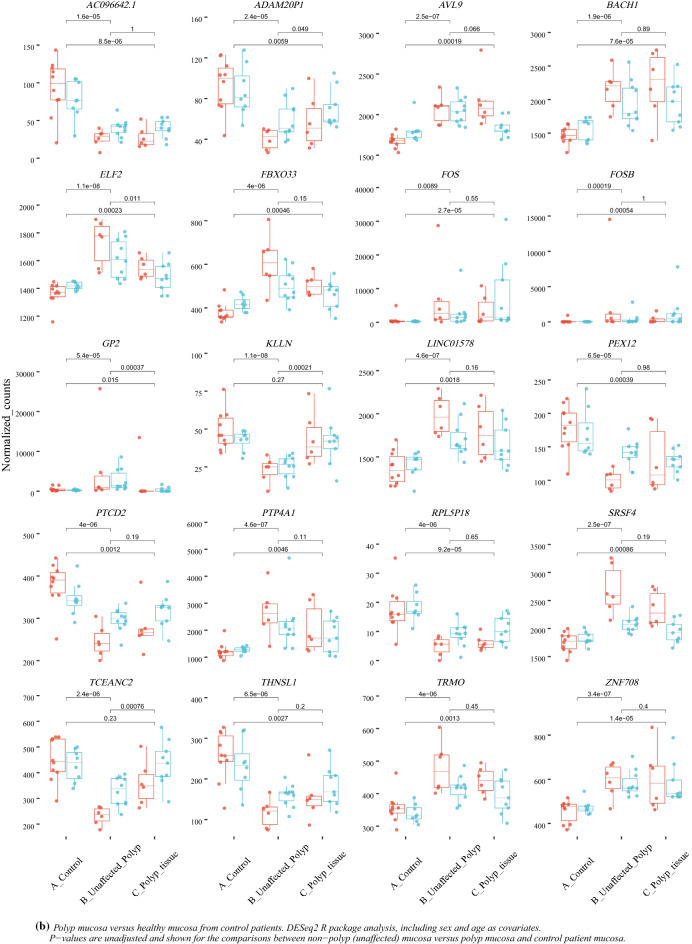
Top 20 differentially expressed genes (DEGs) between non-polyp CRSwNP patient and control patient. (**a**) Polyp mucosa versus non-polyp (unaffected) mucosa from the same CRSwNP patient and (**b**) Healthy control patient mucosa versus non-polyp (unaffected) mucosa from CRSwNP patient. *p*-values are unadjusted and shown for the comparisons between non-polyp mucosa versus polyp mucosa and control patient mucosa. DESeq2 R package analysis, including sex and age as covariates.

**Figure 2:**
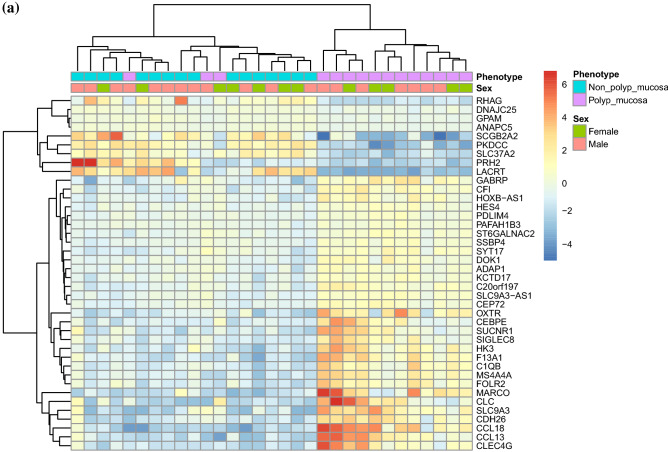

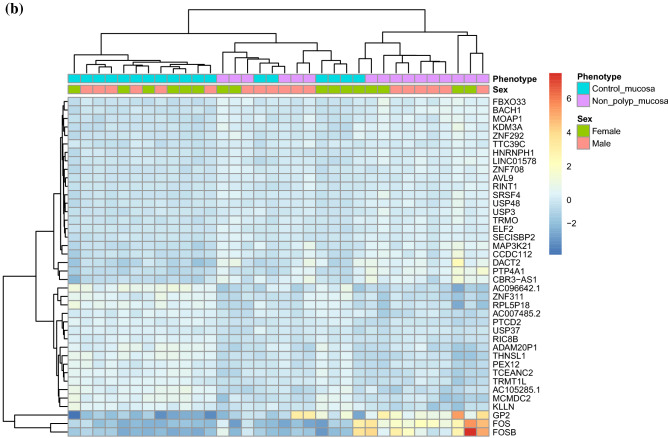
Heatmap of the 40 most differentially expressed genes (DEGs). (**a**) Polyp mucosa versus non-polyp mucosa from the same CRSwNP patient and (**b**) Healthy control patient mucosa versus non-polyp mucosa from CRSwNP patient. DESeq2 R package analysis, including sex and age as covariates. Heatmaps were generated using “pheatmap” in R, R version 4.1.3.

**Figure 3:**
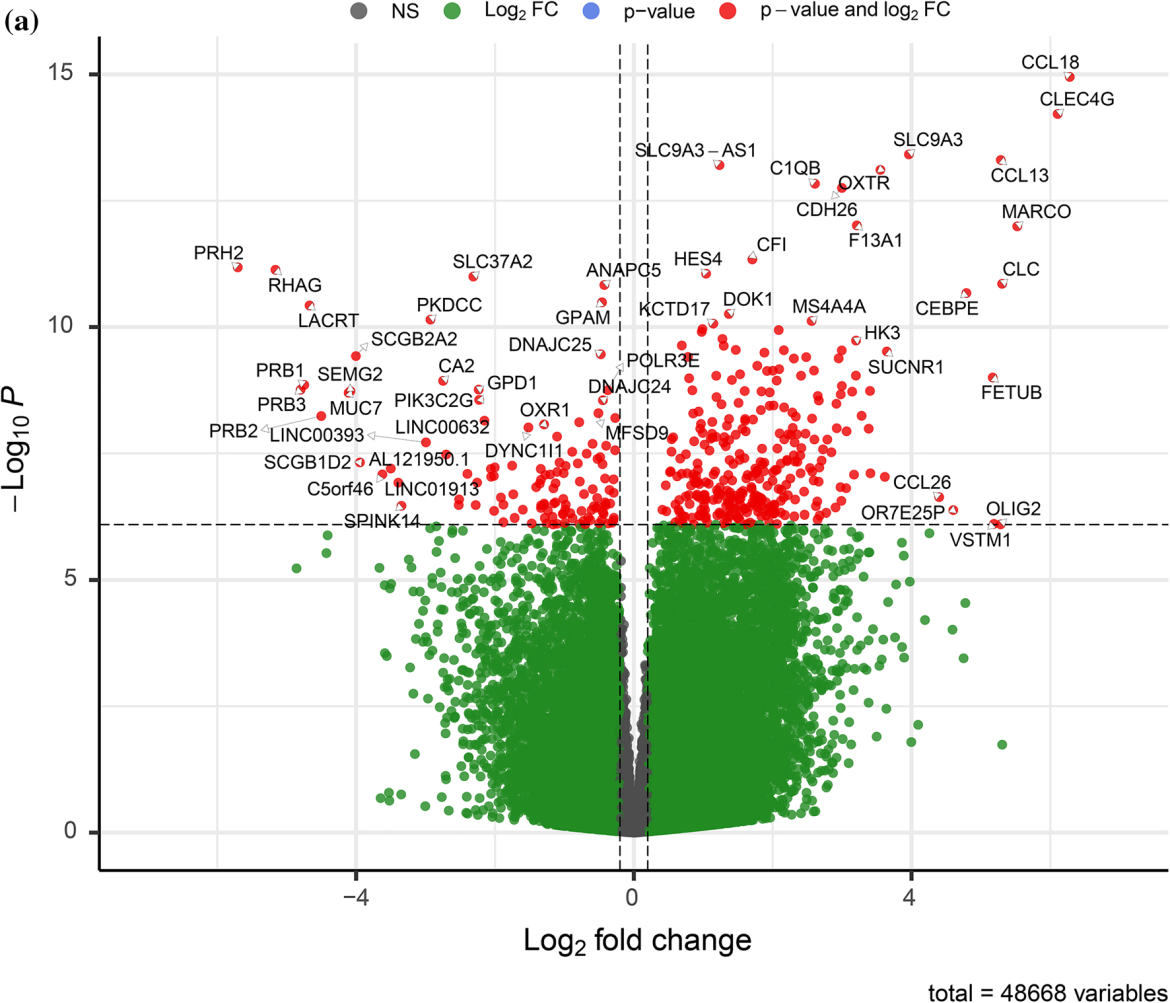

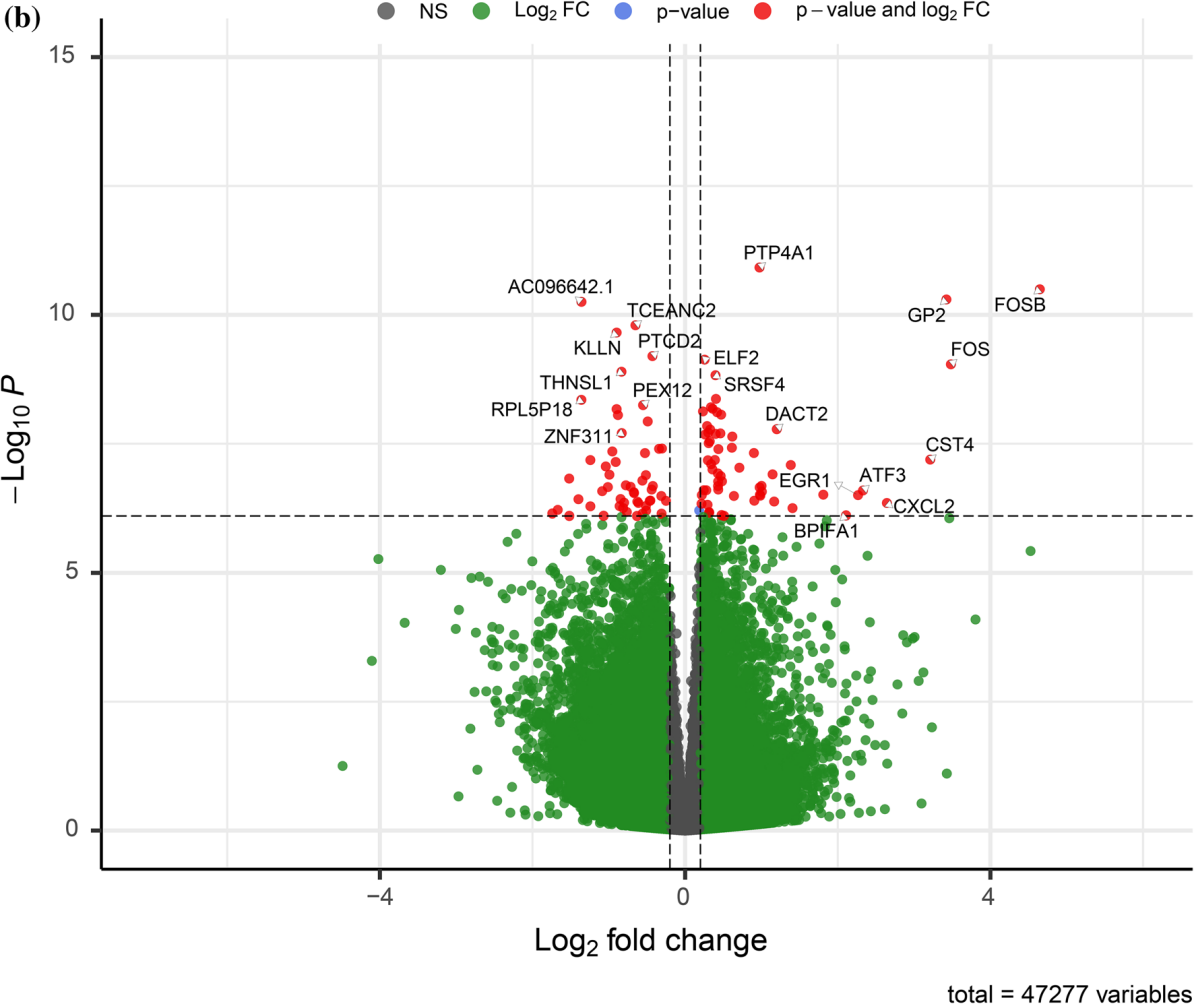
Gene expression of all expressed genes. Vulcano plot showing the negative logarithm of the *p*-value on the y-axis and the fold change of all expressed genes from the comparison of (**a**) Polyp mucosa versus non-polyp mucosa from the same CRSwNP patient and (**b**) Healthy control patient mucosa versus non-polyp mucosa from CRSwNP patient. DESeq2 R package analysis, including sex and age as covariates.

Between polyp mucosa and non-polyp mucosa from the same CRSwNP patient a total of 1968 genes were significantly different using an adjusted *p*-value of < 0.001. The most significant DEGs between polyp mucosa from CRSwNP patients and adjacent non-polyp mucosa from the same CRSwNP patient are shown in Table 1 and Fig. 1a. Results from the over-represented pathway analyses between these two comparisons are shown in Table 2. Top significant DEGs up-regulated in the polyp mucosa include genes involved in T-helper 2 (Th2) type response and eosinophil migration, such as *CCL13*, *CCL18*, *CCL8*^12^. C-Type Lectin Domain Family 4 Member G (*CLEC4G) gene wa*s also up-regulated and represents the second most significant DEG. Comparative analysis of the polyp and non-polyp mucosa from the same patients revealed Solute carrier family 9 member A3 *(SLC9A3) and its antisense gene SLC9A3-AS1* among the top 5 DEGs (Table 1 and Fig. 1a). Additionally, the *HISLA* gene (alias *LINC01146*) that is considered as a main “expression regulatory hub”^17^ were significantly up-regulated in polyp mucosa from CRSwNP patients, albeit also up-regulated in non-polyp mucosa of the same patient compared with healthy mucosa of control individuals. The molecular pathway which was the most statistically significant between polyp mucosa and adjacent non-polyp mucosa of the same patient was “ciliated epithelial cell” (*p* = 8.5 × 10^–78^). Other pathways, which included significant DEGs were “Metabolic pathways” (108 DEGs, adjusted *p* = 0.001), “Microglia Pathogen Phagocytosis Pathway” (16 DEGs, adjusted *p* = 9.97 × 10^–07^, “inflammation mediated by chemokine and cytokine signaling pathway” (24 DEGs, adjusted *p* = 0.003 and “Neutrophil degranulation” (54 DEGs, adjusted *p* = 1.32 × 10^–05^). A heatmap with the top 40 DEGs between polyp mucosa and non-polyp mucosa from the same CRSwNP patient is shown in Fig. 2a. A volcano plot showing the result from all expressed genes is shown in Fig. 3a.

**Table 1:** The 40 top differentially expressed genes (DEGs) in non-polyp nasal epithelial mucosa from patients with CRSwNP versus polyp mucosa in the same patient.

RNA	baseMean	log2FoldChange	lfcSE	stat	*p* value	padj
*CCL18*	335.54	6.28	0.78	− 8.01	1.12 × 10^−15^	3.67 × 10^−11^
*CLEC4G*	75.28	6.11	0.78	− 7.80	6.06 × 10^−15^	9.93 × 10^−11^
*SLC9A3*	460.64	3.96	0.52	− 7.57	3.83 × 10^−14^	4.04 × 10^−10^
*CCL13*	222.07	5.29	0.70	− 7.53	4.94 × 10^−14^	4.04 × 10^−10^
*SLC9A3-AS1*	810.12	1.23	0.16	− 7.50	6.23 × 10^−14^	4.09 × 10^−10^
*OXTR*	385.95	3.55	0.48	− 7.47	7.75 × 10^−14^	4.23 × 10^−10^
*C1QB*	533.27	2.61	0.35	− 7.39	1.46 × 10^−13^	6.82 × 10^−10^
*CDH26*	1632.59	3.00	0.41	− 7.37	1.77 × 10^−13^	7.23 × 10^−10^
*F13A1*	2594.99	3.21	0.45	− 7.13	9.73 × 10^−13^	3.33 × 10^−09^
*MARCO*	186.31	5.52	0.77	− 7.13	1.02 × 10^−12^	3.33 × 10^−09^
*CFI*	786.72	1.71	0.25	− 6.92	4.58 × 10^−12^	1.37 × 10^−08^
*PRH2*	220.91	− 5.71	0.83	6.87	6.58 × 10^−12^	1.80 × 10^−08^
*RHAG*	24.29	− 5.16	0.75	6.85	7.36 × 10^−12^	1.85 × 10^−08^
*HES4*	103.20	1.04	0.15	− 6.82	8.80 × 10^−12^	2.06 × 10^−08^
*SLC37A2*	1936.03	− 2.31	0.34	6.81	1.00 × 10^−11^	2.19 × 10^−08^
*CLC*	251.95	5.31	0.78	− 6.76	1.39 × 10^−11^	2.84 × 10^−08^
*ANAPC5*	3935.01	− 0.43	0.06	6.75	1.47 × 10^−11^	2.84 × 10^−08^
*CEBPE*	23.46	4.79	0.72	− 6.70	2.12 × 10^−11^	3.87 × 10^−08^
*GPAM*	529.09	− 0.46	0.07	6.64	3.23 × 10^−11^	5.57 × 10^−08^
*LACRT*	256.48	− 4.67	0.71	6.61	3.73 × 10^−11^	6.12 × 10^−08^
*DOK1*	171.50	1.37	0.21	− 6.56	5.51 × 10^−11^	8.60 × 10^−08^
*PKDCC*	1217.18	− 2.93	0.45	6.52	7.15 × 10^−11^	1.06 × 10^−07^
*MS4A4A*	223.73	2.56	0.39	− 6.51	7.61 × 10^−11^	1.08 × 10^−07^
*KCTD17*	80.18	1.15	0.18	− 6.49	8.58 × 10^−11^	1.17 × 10^−07^
*ST6GALNAC2*	161.85	0.99	0.15	− 6.45	1.10 × 10^−10^	1.44 × 10^−07^
*HOXB-AS1*	25.15	2.09	0.32	− 6.45	1.15 × 10^−10^	1.44 × 10^−07^
*SSBP4*	441.60	0.98	0.15	− 6.43	1.25 × 10^−10^	1.51 × 10^−07^
*CEP72*	214.53	1.28	0.20	− 6.38	1.72 × 10^−10^	2.01 × 10^−07^
*HK3*	147.80	3.20	0.50	− 6.37	1.85 × 10^−10^	2.10 × 10^−07^
*PAFAH1B3*	89.57	0.69	0.11	− 6.34	2.32 × 10^−10^	2.48 × 10^−07^
*ADAP1*	46.78	1.29	0.20	− 6.34	2.35 × 10^−10^	2.48 × 10^−07^
*SYT17*	148.54	1.47	0.23	− 6.31	2.84 × 10^−10^	2.80 × 10^−07^
*GABRP*	2266.10	2.16	0.34	− 6.31	2.88 × 10^−10^	2.80 × 10^−07^
*SIGLEC8*	39.31	3.00	0.48	− 6.30	2.90 × 10^−10^	2.80 × 10^−07^
*SUCNR1*	68.04	3.64	0.58	− 6.30	3.05 × 10^−10^	2.86 × 10^−07^
*C20orf197*	29.59	1.88	0.30	− 6.29	3.21 × 10^−10^	2.92 × 10^−07^
*DNAJC25*	226.50	− 0.48	0.08	6.28	3.46 × 10^−10^	3.07 × 10^−07^
*SCGB2A2*	369.54	− 4.00	0.64	6.26	3.76 × 10^−10^	3.23 × 10^−07^
*PDLIM4*	296.85	0.78	0.12	− 6.26	3.91 × 10^−10^	3.23 × 10^−07^
*FOLR2*	139.85	2.48	0.39	6.25	3.94 × 10^−10^	3.23 × 10^−07^

The mRNA levels (baseMean) of expressed genes and p-values adjusted using the Benjamini–Hochberg method.

*BaseMean* mean RNA count, *lfcSE* log2 Fold Change Standard Error, *stat* Wald statistic Z-score, *padj p*-value adjusted.

**Table 2:** Enriched pathways among the top 1698 differentially expressed genes (DEGs) from the DESeq2 analysis of polyp versus adjacent non-polyp mucosa in the same CRSwNP patient.

KEGG—pathways	Number of hits	Expected score	Adjusted *p*-value
Metabolic pathways	108	66.14	0.001
Phagosome	24	7.14	0.001
Complement and coagulation cascades	16	3.65	0.002
WikiPathways			
Microglia pathogen phagocytosis pathway	**16**	**1.67**	**9.97 × 10–07**
Genes related to primary cilium development (based on CRISPR)	23	5.11	2.07 × 10^–05^
TYROBP Causal Network	14	2.87	0.003
Human Complement System	16	4.07	0.006
Intraflagellar transport proteins binding to dynein	9	1.30	0.010
PANTHER—pathways			
Inflammation mediated by chemokine and cytokine signaling pathway	24	8.24	0.003
Reactome—pathways			
Neutrophil degranulation	54	21.16	1.32 × 10^–05^
Intraflagellar transport	16	2.50	9.24 × 10^–05^
Anchoring of the basal body to the plasma membrane	18	4.22	0.002
Immunoregulatory interactions (Lymphoid and a non-Lymphoid cell)	17	4.48	0.009
Cell marker/cell types			
Esophagus—Ciliated epithelial cell (425)	**162**	**20.01**	**8.53 × 10**^**–78**^
Lung—Ciliated cell (276)	**122**	**12.72**	**1.49 × 10**^**–64**^
Microglial cell (483)	88	21.58	6.31 × 10^–23^
Paneth cell (314)	69	13.71	3.32 × 10^–22^
CD1C-CD141- dendritic cell (314)	55	14.07	1.93 × 10^–13^
Monocyte (846)	96	37.32	3.33 × 10^–13^
SLC16A7 + cell (988)	105	43.42	5.41 × 10^–13^
Macrophage (105)	25	4.48	1.42 × 10^–08^
Sertoli cell (457)	56	21.00	4.67 × 10^–08^
Eosinophil (54)	16	2.24	1.34 × 10^–06^

Analyzed using Genetrail 3.029 *p*-values adjusted using the Benjamini-yekutieli method.

(Genes *p*-value adjusted < 0.001, n = 1698).

Significant values are in bold.

### Profiling DEGs between mucosa from healthy control vs non-polyp mucosa from patients with CRSwNP

Overall, 1494 RNAs were differentially expressed between healthy mucosa of control vs non-polyp mucosa of patients with CRSwNP (Genes *p*-adjusted < 0.01). The most significant DEGs are shown in Fig. 1b and Table 3. Results from the over-represented pathway analyses between this comparison are shown in Table 4. The most significant pathways were the ones involving natural killer T (NKT) cell (*p* = 2.4 × 10^–45^) and herpes simplex virus 1 (HSV-1) infection (4.3 × 10^–19^). Altered expression of genes related to NKT-cells and HSV-1, may point to the deregulation of viral defense in CRSwNP.

**Table 3:** The 40 top differentially expressed genes (DEGs) in non-polyp nasal epithelial mucosa from patients with CRSwNP versus mucosal tissue from controls.

RNA	baseMean	log2FoldChange	lfcSE	stat	*p*-value	padj
*PTP4A1*	1777.09	0.97	0.14	6.78	1.21 × 10^−11^	3.41 × 10^−07^
*FOSB*	646.71	4.65	0.70	6.64	3.19 × 10^−11^	3.95 × 10^−07^
*GP2*	1981.93	3.42	0.52	6.57	5.03 × 10^−11^	3.95 × 10^−07^
*AC096642*.*1*	61.77	− 1.36	0.21	− 6.55	5.60 × 10^−11^	3.95 × 10^−07^
*TCEANC2*	369.15	− 0.65	0.10	− 6.40	1.59 × 10^−10^	9.00 × 10^−07^
*KLLN*	35.86	− 0.90	0.14	− 6.35	2.21 × 10^−10^	1.04 × 10^−06^
*PTCD2*	322.19	− 0.43	0.07	− 6.18	6.33 × 10^−10^	2.55 × 10^−06^
*ELF2*	1507.39	0.26	0.04	6.16	7.41 × 10^−10^	2.61 × 10^−06^
*FOS*	2302.83	3.48	0.57	6.12	9.14 × 10^−10^	2.87 × 10^−06^
*THNSL1*	196.42	− 0.83	0.14	− 6.07	1.27 × 10^−09^	3.59 × 10^−06^
*SRSF4*	2023.33	0.40	0.07	6.05	1.49 × 10^−09^	3.82 × 10^−06^
*LINC01578*	1582.75	0.40	0.07	5.87	4.28 × 10^−09^	9.69 × 10^−06^
*RPL5P18*	12.89	− 1.36	0.23	− 5.87	4.46 × 10^−09^	9.69 × 10^−06^
*PEX12*	151.12	− 0.55	0.09	− 5.83	5.66 × 10^−09^	1.12 × 10^−05^
*ZNF708*	518.62	0.34	0.06	5.81	6.22 × 10^−09^	1.12 × 10^−05^
*TRMO*	389.92	0.36	0.06	5.80	6.65 × 10^−09^	1.12 × 10^−05^
*ADAM20P1*	71.89	− 0.90	0.15	− 5.80	6.75 × 10^−09^	1.12 × 10^−05^
*AVL9*	1876.16	0.23	0.04	5.78	7.48 × 10^−09^	1.15 × 10^−05^
*BACH1*	1744.81	0.42	0.07	5.77	7.76 × 10^−09^	1.15 × 10^−05^
*FBXO33*	464.49	0.47	0.08	5.76	8.66 × 10^−09^	1.18 × 10^−05^
*AC105285*.*1*	50.92	− 0.88	0.15	− 5.75	8.81 × 10^−09^	1.18 × 10^−05^
*TRMT1L*	658.27	− 0.49	0.09	− 5.70	1.17 × 10^−08^	1.50 × 10^−05^
*SECISBP2*	2027.42	0.29	0.05	5.67	1.44 × 10^−08^	1.77 × 10^−05^
*DACT2*	133.52	1.20	0.21	5.64	1.66 × 10^−08^	1.90 × 10^−05^
*USP3*	1582.27	0.33	0.06	5.64	1.68 × 10^−08^	1.90 × 10^−05^
*HNRNPH1*	10 575.10	0.30	0.05	5.62	1.93 × 10^−08^	1.98 × 10^−05^
*ZNF311*	80.44	− 0.83	0.15	− 5.61	1.97 × 10^−08^	1.98 × 10^−05^
*KDM3A*	2057.25	0.46	0.08	5.61	2.00 × 10^−08^	1.98 × 10^−05^
*ZNF292*	3415.61	0.40	0.07	5.61	2.04 × 10^−08^	1.98 × 10^−05^
*RINT1*	517.18	0.26	0.05	5.60	2.11 × 10^−08^	1.98 × 10^−05^
*MAP3K21*	260.50	0.62	0.11	5.59	2.30 × 10^−08^	2.10 × 10^−05^
*USP48*	2054.50	0.32	0.06	5.55	2.84 × 10^−08^	2.51 × 10^−05^
*TTC39C*	962.55	0.31	0.06	5.54	3.09 × 10^−08^	2.64 × 10^−05^
*CCDC112*	216.29	0.61	0.11	5.50	3.79 × 10^−08^	3.07 × 10^−05^
*USP37*	1073.22	− 0.30	0.06	− 5.49	3.93 × 10^−08^	3.07 × 10^−05^
*RIC8B*	498.24	− 0.34	0.06	− 5.49	3.98 × 10^−08^	3.07 × 10^−05^
*MOAP1*	463.38	0.44	0.08	5.49	4.03 × 10^−08^	3.07 × 10^−05^
*MCMDC2*	44.72	− 0.95	0.17	− 5.47	4.47 × 10^−08^	3.32 × 10^−05^
*CBR3-AS1*	293.55	0.90	0.17	5.46	4.80 × 10^−08^	3.40 × 10^−05^
*AC007485*.*2*	78.67	− 0.53	0.10	− 5.46	4.81 × 10^−08^	3.40 × 10^−05^

The mRNA levels (baseMean) of expressed genes and p-values adjusted using the Benjamini–Hochberg method.

*BaseMean* mean RNA count, *lfcSE* log2 Fold Change Standard Error, *stat* Wald statistic Z-score, *padj p*-value adjusted.

**Table 4:** Enriched pathways among the top 1494 differentially expressed genes (DEGs) from the DESeq2 analysis of healthy mucosa from control vs non-polyp mucosa from patient with CRSwNP.

KEGG—pathways	Number of hits	Expected score	Adjusted p-value
Herpes simplex virus 1 infection (Genes pcorr < 0.05, n = 3863)	**103**	**29.69**	**4.33 × 10**^**–19**^
Herpes simplex virus 1 infection (Genes pcorr < 0.01, n = 1494)	44	11.48	1.46 × 10^–09^
Protein processing in endoplasmic reticulum	27	6.11	1.17 × 10^–06^
Cell cycle	21	4.72	4.50 × 10^–05^
Systemic lupus erythematosus	20	5.24	5.72 × 10^–04^
WikiPathways			
Cell Cycle	21	4.55	8.88 × 10^–05^
Circadian rhythm related genes	23	7.58	0.005
PANTHER—pathways			
N/A			
Reactome—pathways			
Generic Transcription Pathway	36	7.02	1.86 × 10^–10^
HATs acetylate histones	29	5.85	4.94 × 10^–08^
Estrogen-dependent gene expression	28	5.68	7.87 × 10^–08^
Meiotic recombination	22	3.55	1.57 × 10^–07^
Cell marker/cell types			
Natural killer T (NKT) cell (4557)	**377**	**171.92**	**2.35 × 10**^**–45**^
Meiotic prophase fetal germ cell (1038)	86	41.12	2.50 × 10^–07^
Neural progenitor cell (175)	27	7.02	4.24 × 10^–06^
SLC16A7 + cell (988)	72	36.09	1.69 × 10^–05^
Mitotic arrest phase fetal germ cell (951)	69	37.17	2.69 × 10^–04^
Naive CD8 + T cell (93)	14	3.47	0.003

Analyzed using GeneTrail 3.0^30^. *p*-values adjusted using the Benjamini-yekutieli method.

(Genes *p*-value adjusted < 0.01, n = 1494).

Significant values are in bold.

A heatmap with the top 40 DEGs between healthy mucosa of control vs non-polyp mucosa of patients with CRSwNP is shown in Fig. 2b. A volcano plot showing the result of all expressed genes is shown in Fig. 3b. Top significant up-regulated DEGs were *PTP4A1*, *GP2*, *ELF2*, *FOSB* and *FOS*. Additionally, *AC096642*.*1*, *TCEANC2*, *KLLN and PTCD2* were among the most significantly down-regulated genes in polyp mucosa.

### Profiling DEGs between healthy mucosa from control vs polyp mucosa from patients with CRSwNP

Between polyp mucosa and healthy mucosa from control patients, a total of 1733 genes were significantly differentially expressed (adjusted *p*-value of < 0.001). In Supplementary Table S1 and supplementary Fig. S1, the most significant DEGs between polyp mucosa from CRSwNP patients and healthy mucosa from the same CRSwNP patient are shown. The most significant pathways from the over-represented analyses were, considering the comparison within the same CRSwNP patient, the ones involving cilium processes and cilium cellular components (*p* = 7.1 × 10^–32^). Out of the ten most associated genes (Supplementary Table S1 and Fig. S1), seven were also among the top associated genes when comparing healthy mucosa and polyp tissue from the same patient (Table 1, Fig. 1a).

### Principal component analysis (PCA)

**Figure 4:**
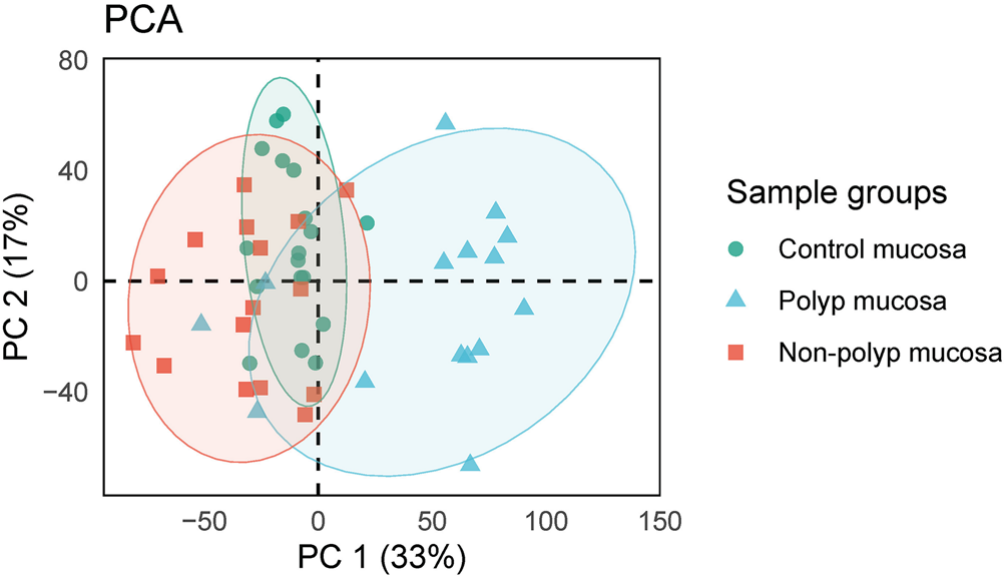
Plot showing the principal components analysis (PCA) grouped by healthy control patient mucosa, non-polyp and polyp mucosa from CRSwNP patient.

The distribution of samples on the PC1–PC2 planes visualize the difference between healthy control and CRSwNP samples and if gene expression can be used to correctly classify the patients with CRSwNP. A PCA plot showing the overall relationship of the three tissue types investigated in this study, CRSwNP-polyp, CRSwNP-adjacent non-polyp tissue and healthy control tissue is shown in Fig. 4. PCA plots of sex and age are included in the supplementary information, Supplementary Figs. S3 and S4.

### DEGs associated with COVID-19

We also analyzed genes specifically located in four chromosomal regions on chromosome 3, 12, 19 and 21, which show genome wide significant association with COVID-19.

**Figure 5:**
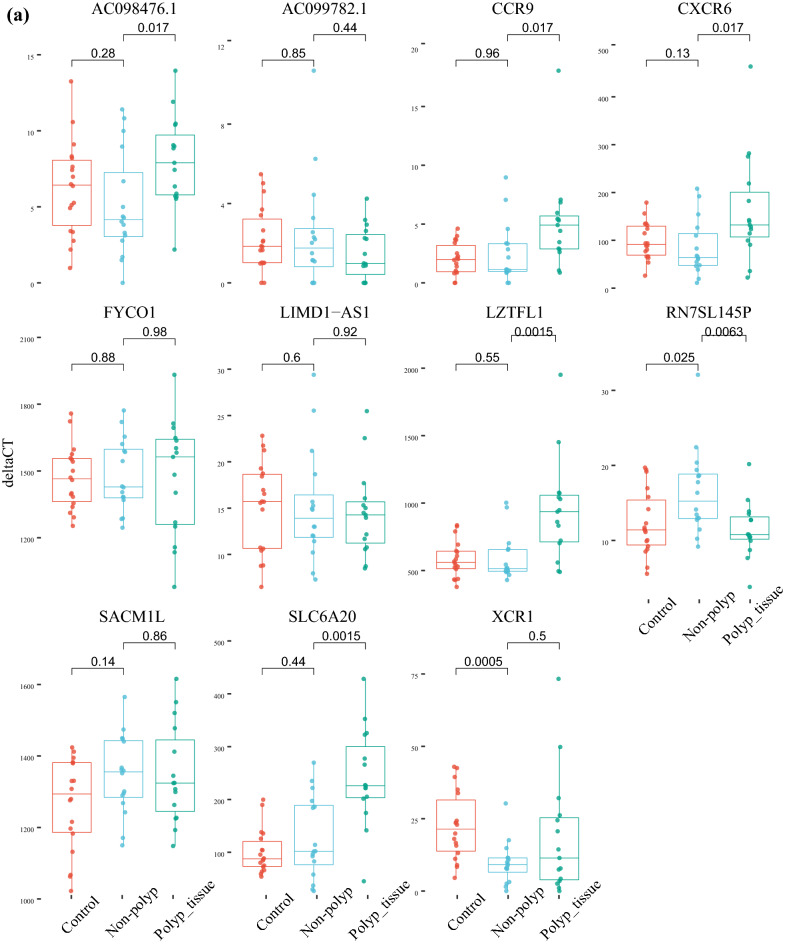

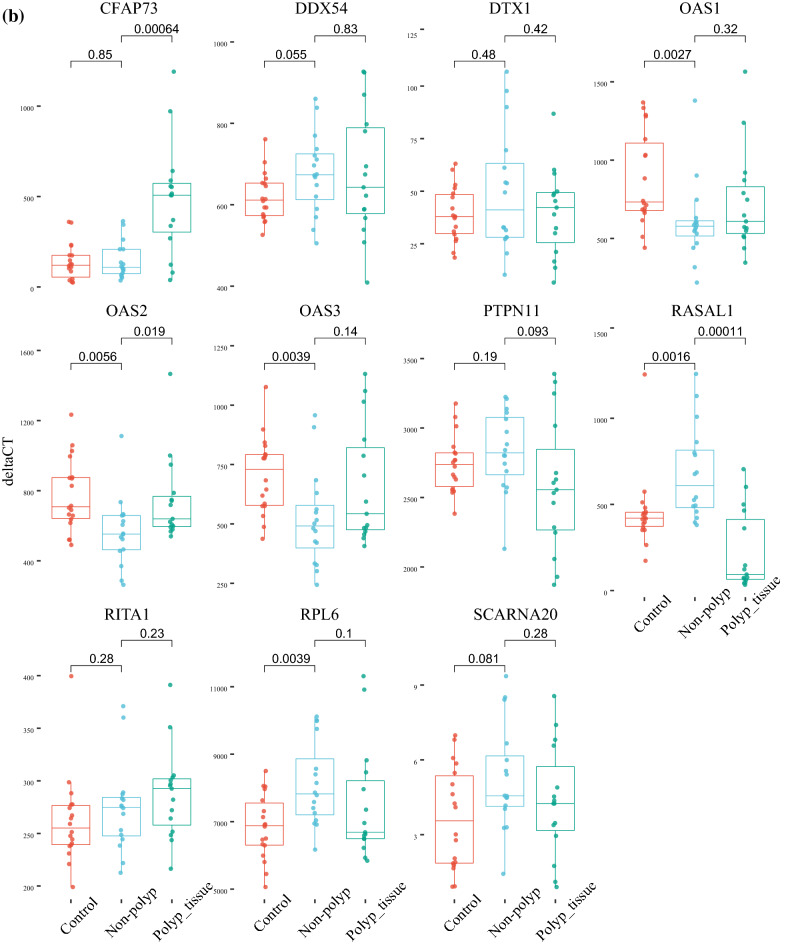

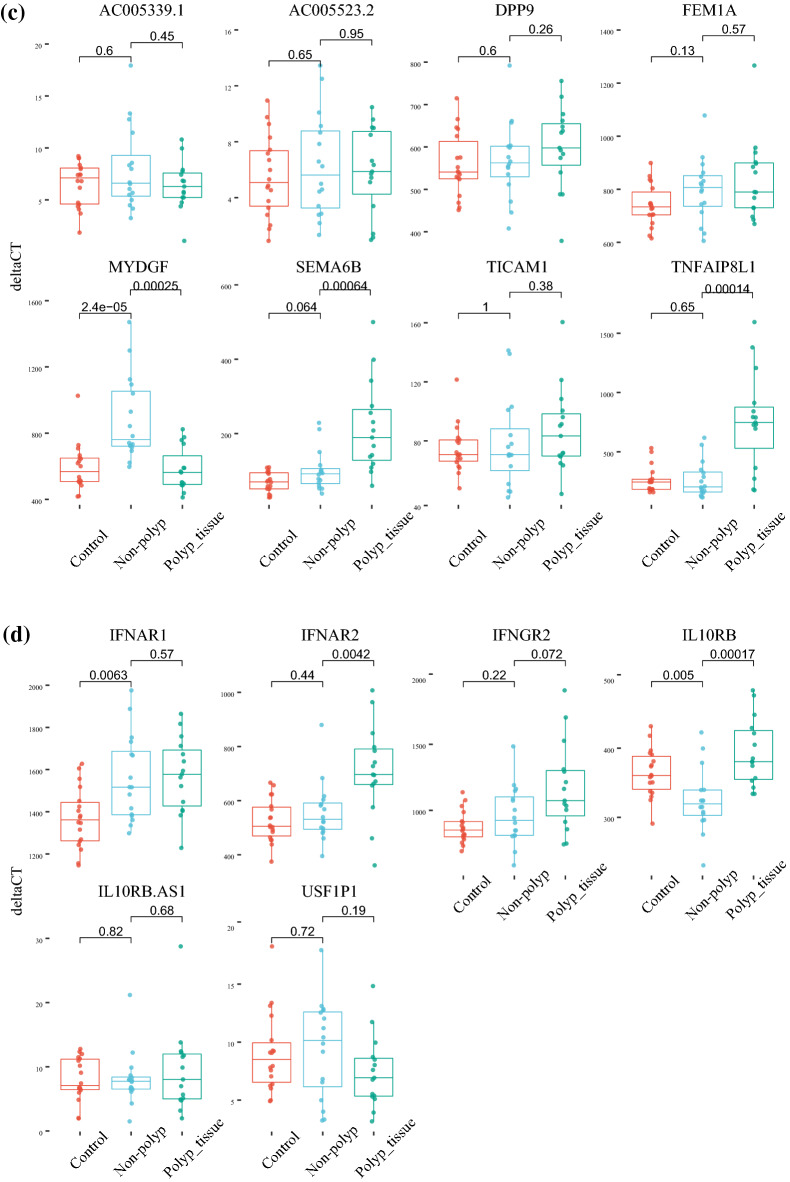
Differentially expressed genes (DEGs) in chromosomal regions showing genome wide significant association with COVID-19. Four chromosomal regions were included, (**a**) chromosome 3, (**b**) chromosome 12, (**c**) chromosome 19 and (**d**) chromosome 21.

The significant DEGs in nasal mucosa within these gene-regions were the genes *IFNAR1*, *IFNAR2* and *IL10RB* located on chromosome 21, *LZTFL1*, *CCR9*, *RN7SL145P*, *SLC6A20* and *XCR1* located on chromosome 3, *MYDGF SEMA6B* and *TNFAIP8L1* located on chromosome 19 and *CFAP73*, *OAS1*, *OAS2*, *OAS3* and *RASAL1* on chromosome 12 (Fig. 5a–d).

The gene *LZTFL1* is involved in cilia inhibition, and shows a higher expression in polyp mucosa of CRSwNP patients. Overall, we can show that genes involved in cilia formation are dramatically altered in nasal polyp tissue from patients with CRSwNP compared with non-polyp tissue from the same patient as well as from control patients.

### DEGs associated with the immune system

Next, we analyzed our DEGs and pathways involved in the immune system. Several pathway databases indicated genes involved in “Complement system and coagulation cascades” (genes: *ARRB2*, *C3*, *C3AR1*, *C5AR1*, *C6*, *CFI*, *CR1*, *F12*, *F13A1*, *FCGR3A*, *GNA15*, *GNAI2*, *IBSP*, *LRP2*, *SELPLG*, *VSIG4*, *C1QA*, *C1QB*, *C1QC*, *SERPINA1*, *SERPINF2*. Genes involved in “Neutrophil degranulation” were also among the top over-represented including the following 54 genes: *ADAM8*, *ADGRE3*, *ADGRG3*, *AGA*, *ALDH3B1*, *AMPD3*, *BST2*, *C3*, *C3AR1*, *C5AR1*, *CEP290*, *CLEC12A*, *CNN2*, *CPPED1*, *CR1*, *CRACR2A*, *CRISP3*, *CTSC*, *CTSS*, *CYBA*, *CYBB*, *DNAJC3*, *DYNLL1*, *DYNLT1*, *FCER1G*, *FCGR2A*, *FTL*, *GALNS*, *GUSB*, *HEXB*, *HK3*, *HP*, *ITGAM*, *LAIR1*, *LAMP2*, *MMP25*, *MS4A3*, *MVP*, *NEU1*, *OSCAR*, *PLAC8*, *PTAFR*, *RAB37*, *RAB44*, *RNASE2*, *SERPINA1*, *SERPINB10*, *SLC27A2*, *SLPI*, *STING1*, *STK11IP*, *STOM*, *TUBB4B*, *TYROBP*. In addition, genes involved in “inflammation mediated by chemokine and cytokine signaling” were significantly over-represented including: *ACTG1*, *ADCY6*, *ALOX15*, *ALOX5AP*, *ARPC1B*, *C5AR1*, *CCL13*, *CCL18*, *CCL26*, *CCR1*, *CCR3*, *GNA15*, *GNAI2*, *GNG4*, *ITPR1*, *MYLK3*, *MYO3A*, *PLCB1*, *PLCB2*, *PTGS1*, *RHOC*, *SOCS6*, *SOCS7*, *VAV1*.

*Other genes involved in cytokine signaling that were among the most differentially expressed*, *but not included in an over represented network*, *were ACKR3*, *BMPR1A*, *BMPR1B*, *CCL15*, *CSF1R*, *CSF3*, *IL10*, *IL13RA1*, *IL1RAP*, *IL1RL1*, *IL2RA*, *IL37*, *IL5RA*, *IL6R*, *IL9R*, *TNFRSF19*, *TNFSF18* (Supplementary Fig. S2).

### The impact of gender and age on the transcriptomics profile of nasal mucosa in healthy controls

A total of 1683 genes were significantly differently expressed by age using gender as a covariate, while gender had a significant influence on the expression of 1313 genes (*p* < 0.05) when correcting for age.

Since age and sex influence the expression of a substantial proportion of genes, we use them as covariates in order to identify differential expressed genes regardless of the influence of age or sex.

## Discussion

Using whole transcriptomics analyses with a relatively large sample size of 50 nasal mucosa biopsies, this study has expanded our current knowledge of the transcriptome profile of polyp mucosa of CRSwNP patients integrating sex and age as covariates in the analysis.

Our results confirm, and expand, previous studies suggesting the induction of genes involved in inflammation and the dysregulation of genes involved in cilia generation in polyp mucosa^12–19^. We found that the expression of the cilia inhibitory gene *LZTF1*, located in the most associated gene region of COVID-19, is up-regulated in polyp mucosa, supporting a potential common mechanism in the pathogenesis of CRSwNP and viral infections like COVID-19. Previous studies have shown that loss of cilia related proteins cause anosmia^32^ a common symptom of COVID-19^33^, and coronaviruses selectively target ciliated cells, and can lead to the withdrawal of the cilia into the cell body, suggesting that the loss of cilia is likely to cause rhinorrhoea.^34^.

The second most up-regulated gene in polyp mucosa was a member of C-type lectin protein family called *CLEC4G* (alias: LSECtin). This protein interacts with surface glycoproteins from several viruses including the spike protein of SARS coronaviruses^35^. CLEC4G has been shown to bind directly to spike's N-terminal domain of SARS-CoV-2 and allow entry in an ACE2-independent fashion^36^.

In non-polyp mucosa of CRSwNP patients, we have additionally identified DEGs associated with NKT-cell and HSV-1 infection pathways. The most differentially expressed gene was however the protein tyrosine phosphatase 4a1 (PTP4A1). PTP4A1 is believed to play a role in the development and maintenance of differentiating epithelial tissues and enhances cell proliferation, cell motility and invasive activity, and promotes cancer metastasis (UniProt Q93096)^37^. PTP4A1 is higher expressed in non-polyp mucosa from CRSwNP patients compared to healthy controls, as well as in tumours and is strongly down-regulated upon tetrodotoxin treatment^38^.

In the pathway enrichment analysis, HSV-1 infection was the top Kegg pathway identified when controls were compared with non-polyp mucosa from CRSwNP patients. Feng Lan et al. has suggested that the inadequate response of CRSwNP may be associated with a deeper intrusion of viruses^39^ and, epithelial damage. Intrusion of HSV-1 into nasal mucosa has been shown to be more extensive in nasal polyp tissue compared to that of non-CRS controls^40^. Nasal polyp tissue may thereby be more susceptible to virus invasion and studies have reported a higher prevalence of respiratory viruses in the nasal mucosa of CRS patients compared to that of controls^41,42^. Additionally, a study by Zaravinos et al.^43^ has shown an increase in the prevalence of human papilloma virus and human herpes virus types 1–7 in human nasal polyposis and that the presence of these viruses likely influences the pathogenesis of the benign nasal tumors. In line with this notion, the transcriptomics study by Peng et al.^19^ found that the top GO sets enriched in non-polyp mucosa from patients with CRSwNP compared with healthy controls were the interferon signalling pathway and pathways involved in viral responses.

The upper airways are specialized in removing airborne pathogens and allergens via effective mucociliary clearance, which is essential to protect the airways from pathogenic insults and prevent pulmonary injury. Ciliary dysfunction may also contribute to the pathogenesis of chronic airway inflammatory diseases such as asthma, allergic rhinitis and CRS, possibly due to the negative impacts of chronic inflammation on mucociliary clearance^44^. Our analysis suggest that gene pathways related to ciliated epithelial cells are significantly altered in nasal polyp tissue, in line with previous observations which show aberrant ciliary marker expression and mis-localization of ciliogenesis markers in CRSwNP patients. This includes the study by Peng et al., 2018^45^ that showed the regulator of motile cilia formation FOXJ1 is mis-localized in CRSwNP patients, which correlated with disease severity and the co-existence of allergic rhinitis or asthma. Further, the ciliary ultrastructural marker DNAH5 is reported to be mis-localized in patients with nasal polyps and that the negative modulator of ciliogenesis cp110 is upregulated in CRSwNP patients^46,47^.

Importantly, our study revealed that the gene *LZTFL1*, which is involved in cilia inhibition and located in the most associated gene region of COVID-19^48^, shows an upregulated expression in polyp tissue from CRSwNP patients. *LZTFL1* interacts with a Bardet-Biedl syndrome (BBS) protein complex known as the BBSome, and negatively regulate ciliary trafficking of this complex^49^, and similarly to cp110, negatively influence ciliogenesis. BBS proteins are vital to maintain ciliary function by mediating protein trafficking to cilia, and ciliary dysfunction has been implicated in at least 35 different diseases which collectively affect nearly all organ systems^50^. Higher expression of *LZTFL1* in polyp tissue thereby suggests down-regulation of cilia formation in this tissue. However, the mucosa next to the polyps in CRSwNP patients have no different expression of this gene compared to controls.

In vertebrates, cilium-dependent signalling orchestrates important developmental pathways, such as limb development and kidney morphogenesis, and is required for vision, hearing, and smell^51^. Cilium dependent signalling also appears to play a role in obesity^52^ and type II diabetes, which have a high incidence amongst patients with compromised cilia function^53^. Endothelial cells, which are ciliated, have been implicated in flow-sensing and vascular hypertension, intracranial blood vessel formation, and atherosclerosis prevention^54–56^. Recent studies have further unveiled a novel role of primary cilium in preventing vascular regression^57^ where there seems to be a mechanosensory role of primary cilia in vascular hypertension and atherosclerosis. Obesity, type II diabetes and vascular hypertension are all associated with an increased risk of developing a severe illness from COVID-19^58,59^.

Loss of function of ciliary proteins in mice has been shown to affect the olfactory epithelium, causing severe reduction of the ciliated border^31^ and as previously mentioned, clinical manifestation suggested to arise from ciliary defects includes anosmia^31^. Secretory and ciliated cells also contain the highest fraction of SARS-CoV-2-infected cells^60^, and a recent study reported that SARS-CoV-2 preferentially replicates in ciliated cells, damages the ciliary layer, which may result in an impaired mucociliary clearance^61^. Ciliary dysfunction is an emerging theme in COVID-19 pathogenesis and given that ciliated epithelial cells is the most differentially regulated pathway in CRSwNP patients, this could suggest that CRSwNP patients may have an altered susceptibility to be infected with SARS-CoV-2.

Taken together, cilium function is altered in nasal polyp tissue and could potentially be an important component of the ability of virus to infect cells after initial contact. Withdrawal of cilia at the cell surface could be a defense mechanism leading the loss of smell as well as fewer infected cells upon viral contact. Polyp formation could therefore perhaps be part of a faulty response or perhaps over exaggerated response to a perceived or real viral threat.

A strength of this study is using a relatively large number of samples from both nasal polyp and non-polyp tissues obtained from the same patients as well as being able to compare with healthy control mucosa. However, this study has a few limitations. Firstly, due to lack of blood samples from the patients we only evaluated upper airway inflammation, but not systemic inflammation (i.e. eosinophilic versus non-eosinophilic) via blood sampling. Secondly, all CRSwNP patients participating in our study were treated with corticosteroids locally in their nasal cavity, which could serve as a confounding factor. A study by Benson et al.^62^ investigated the expression of over 20,000 genes in nasal polyps before and after glucocorticoid treatment and found that the largest functional group of DEGs included genes relating to inflammation (including immunoglobulin production; antigen processing and presentation; the chemoattraction and activation of granulocytes). Therefore, it is likely that local corticosteroid treatment has influenced our transcriptomics results on the nasal polyp of CRSwNP patients.

In conclusion, we identified genes related to primary cilium development to be the most significantly altered molecular pathway in CRSwNP polyp tissue along with pathways of inflammation and metabolism. One of the cilia genes, *LZTFL1*, is located in the most associated gene region of COVID-19. Given that the nasal mucosa is the first point of infection for many viral diseases including COVID-19, the results from this study suggest overlapping molecular mechanisms underlying the pathogenesis of CRSwNPs and COVID-19. The de-regulation of cilia function in the polyp mucosa during CRSwNP could be a putative biomarker of an abnormal viral defense reaction in CRSwNP and presumably in other diseases affected by cilia withdrawal. The results presented herein enhance our understandings of the transcriptome profile in polyp mucosa and non-polyp mucosa of CRSwNPs patients, and as such may have implications for development of novel interventions strategies to counter the development of CRSwNPs.

## Supplementary Information

Supplementary Figure S1.

Supplementary Figure S2.

Supplementary Figure S3.

Supplementary Figure S4.

Supplementary Table S1.
