# Supplementary material for: Transcriptomics unravels molecular changes associated with cilia and COVID-19 in chronic rhinosinusitis with nasal polyps
Source: Sci Rep. 2023 Apr 21;13:6592. doi: 10.1038/s41598-023-32944-3 (PMC10121071; doi:10.1038/s41598-023-32944-3)
Supplement: Supplementary file 1 — Supplementary Figure S1. [file 41598_2023_32944_MOESM1_ESM.pdf]

FIGURE S1: Top 20 differentially expressed genes (DEGs) between polyp mucosa in CRSwNP patients and healthy controls

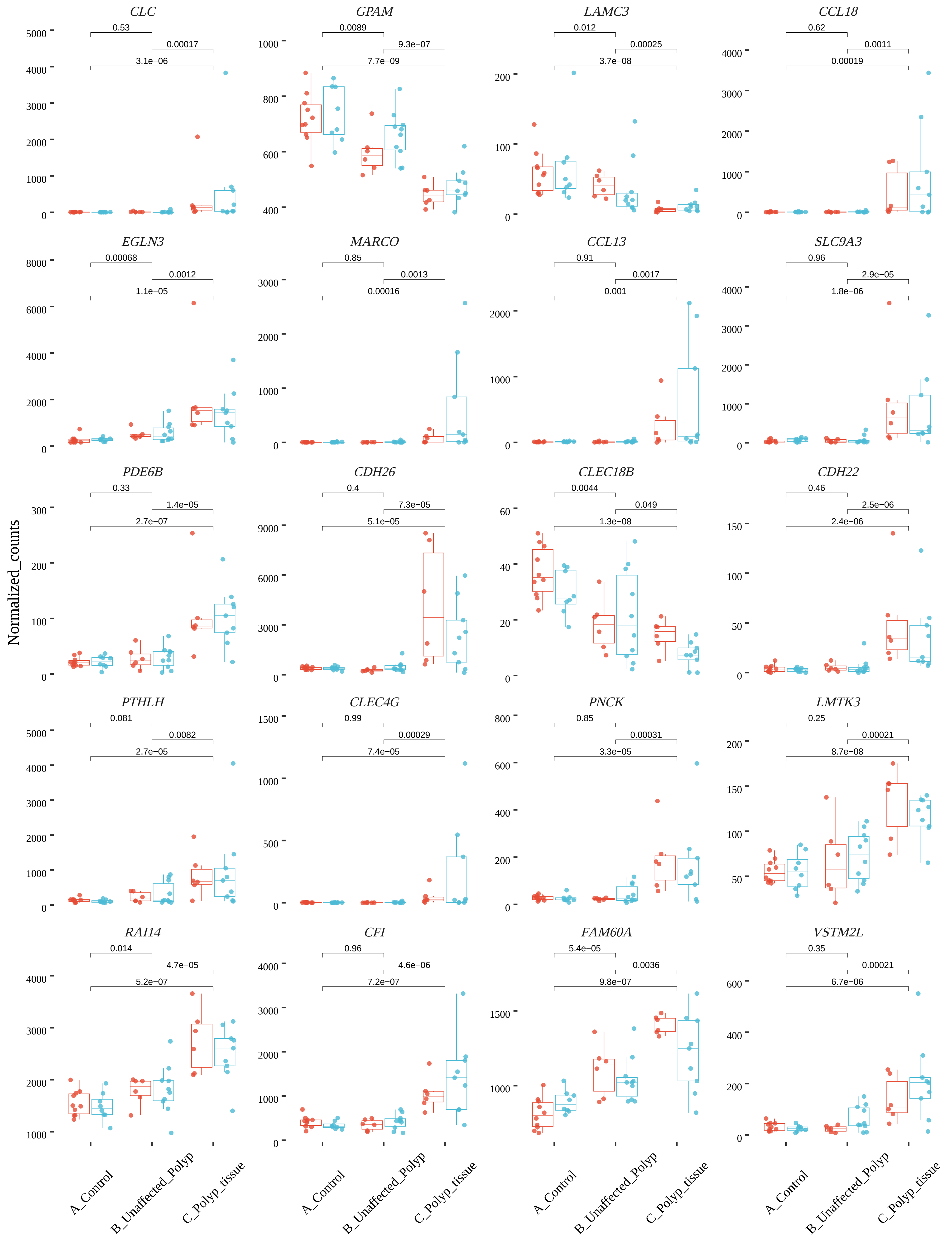

Polyp mucosa versus healthy mucosa from control patients. DESeq2 R package analysis, including sex and age as covariates.
P−values are unadjusted and shown for the comparisons between non−polyp (unaffected) mucosa versus polyp mucosa and control patient mucosa.
