# Supplementary figures and images for: Transcriptomics unravels molecular changes associated with cilia and COVID-19 in chronic rhinosinusitis with nasal polyps
Source: Sci Rep. 2023 Apr 21;13:6592. doi: 10.1038/s41598-023-32944-3 (PMC10121071; doi:10.1038/s41598-023-32944-3)

FIGURE S2. Differentially expressed cytokines in polyp mucosa

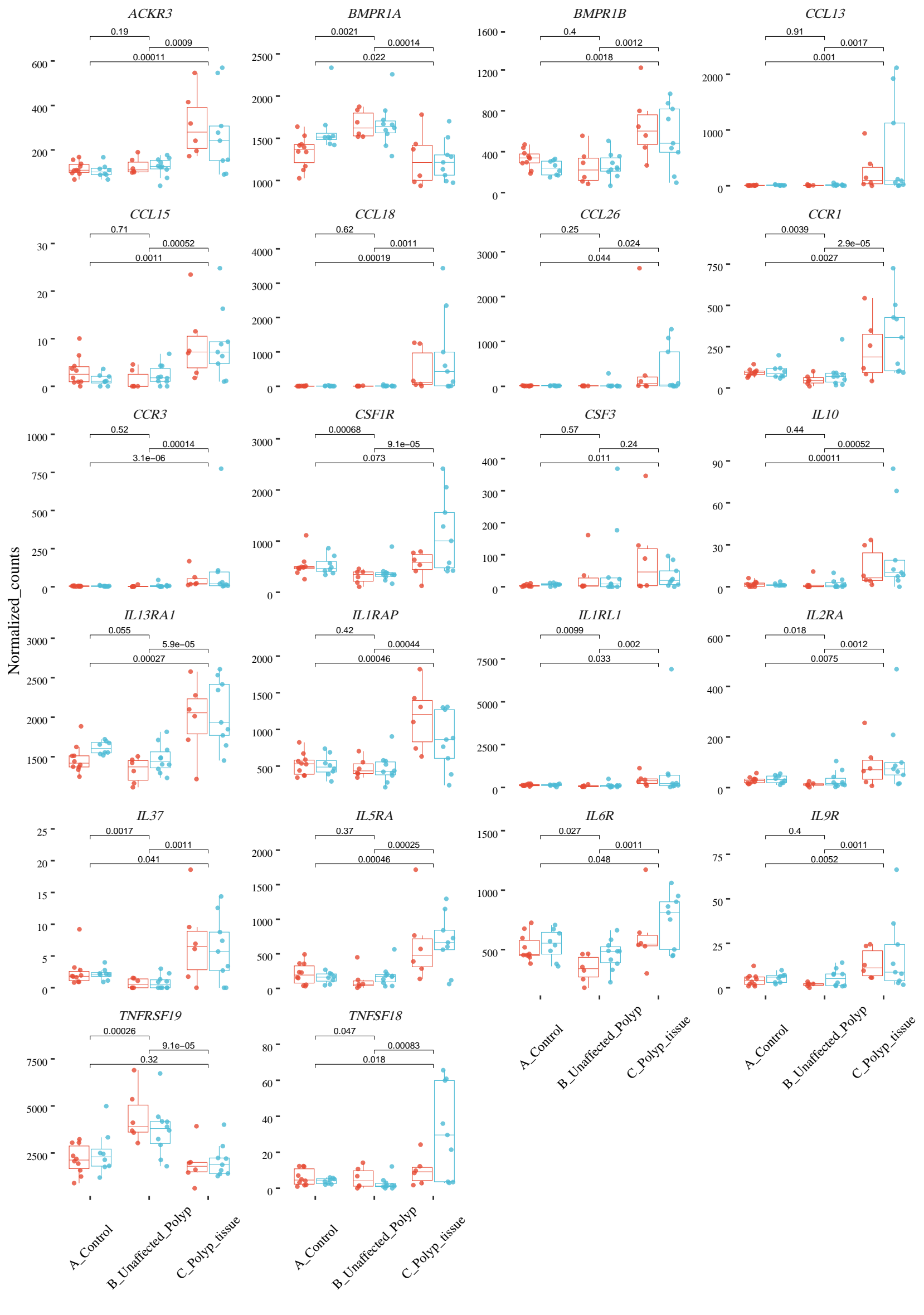

Supplement: Supplementary file 2 — Supplementary Figure S2. [file 41598_2023_32944_MOESM2_ESM.pdf]

**FIGURE S3. Plot showing the principal components analysis (PCA)**  
Grouped by sex.

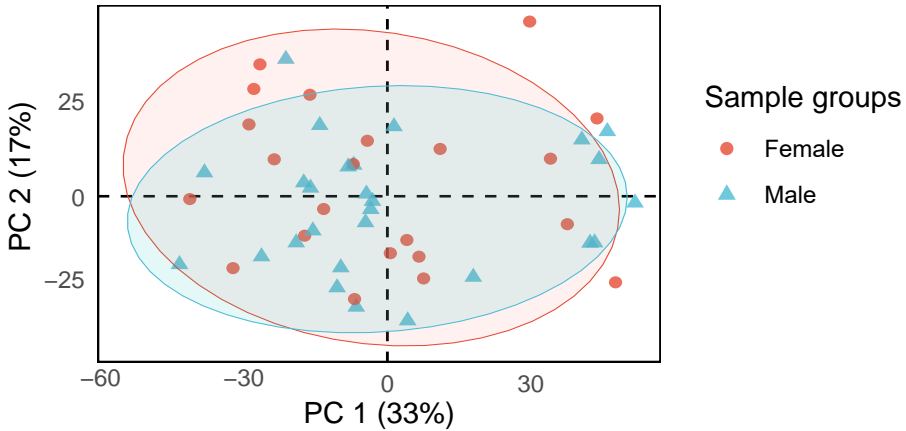

Supplement: Supplementary file 3 — Supplementary Figure S3. [file 41598_2023_32944_MOESM3_ESM.pdf]

**FIGURE S4: Plot showing the principal components analysis (PCA)**  
Grouped by age.

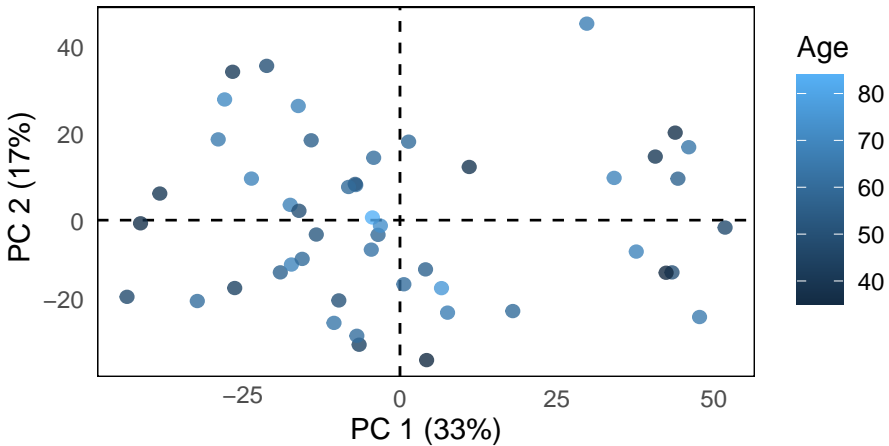

Supplement: Supplementary file 4 — Supplementary Figure S4. [file 41598_2023_32944_MOESM4_ESM.pdf]
