# Supplementary material for: Transcriptomics unravels molecular changes associated with cilia and COVID-19 in chronic rhinosinusitis with nasal polyps
Source: Sci Rep. 2023 Apr 21;13:6592. doi: 10.1038/s41598-023-32944-3 (PMC10121071; doi:10.1038/s41598-023-32944-3)
Supplement: Supplementary file 5 — Supplementary Table S1. [file 41598_2023_32944_MOESM5_ESM.docx]

**Supplementary TABLE 1: The 40 top differentially expressed genes (DEGs) in non-polyp nasal epithelial mucosa from patients with CRSwNP versus polyp mucosa in the same patient.** The mRNA levels (baseMean) of expressed genes and p-values adjusted using the Benjamini-Hochberg method.

| **RNA** | **baseMean** | **log2FoldChange** | **lfcSE** | **stat** | **p-value** | **padj** |
| --- | --- | --- | --- | --- | --- | --- |
| CLC | 255.61 | 6.74 | 0.66 | 10.20 | 1.92E-24 | 6.53E-20 |
| GPAM | 634.15 | -0.66 | 0.07 | -9.42 | 4.66E-21 | 7.93E-17 |
| LAMC3 | 40.67 | -2.93 | 0.31 | -9.29 | 1.48E-20 | 1.68E-16 |
| CCL18 | 344.12 | 6.53 | 0.71 | 9.19 | 3.88E-20 | 3.30E-16 |
| EGLN3 | 966.44 | 2.71 | 0.30 | 8.92 | 4.57E-19 | 3.12E-15 |
| MARCO | 189.86 | 5.74 | 0.66 | 8.63 | 5.99E-18 | 3.40E-14 |
| CCL13 | 227.13 | 5.47 | 0.64 | 8.56 | 1.11E-17 | 5.41E-14 |
| SLC9A3 | 469.29 | 4.16 | 0.49 | 8.45 | 2.86E-17 | 1.22E-13 |
| PDE6B | 62.17 | 2.28 | 0.27 | 8.28 | 1.21E-16 | 4.59E-13 |
| CDH26 | 1695.78 | 3.00 | 0.36 | 8.26 | 1.42E-16 | 4.85E-13 |
| CLEC18B | 24.19 | -1.64 | 0.20 | -8.08 | 6.64E-16 | 2.06E-12 |
| CDH22 | 21.47 | 3.65 | 0.46 | 8.01 | 1.14E-15 | 3.24E-12 |
| PTHLH | 514.21 | 2.97 | 0.37 | 7.98 | 1.47E-15 | 3.85E-12 |
| CLEC4G | 77.09 | 6.18 | 0.78 | 7.94 | 1.96E-15 | 4.78E-12 |
| PNCK | 99.66 | 2.79 | 0.35 | 7.91 | 2.60E-15 | 5.91E-12 |
| LMTK3 | 89.77 | 1.16 | 0.15 | 7.83 | 4.95E-15 | 1.05E-11 |
| RAI14 | 2100.27 | 0.80 | 0.10 | 7.81 | 5.94E-15 | 1.19E-11 |
| CFI | 826.61 | 1.71 | 0.22 | 7.64 | 2.17E-14 | 4.11E-11 |
| FAM60A | 1095.81 | 0.63 | 0.08 | 7.61 | 2.73E-14 | 4.89E-11 |
| VSTM2L | 102.21 | 2.50 | 0.33 | 7.60 | 2.99E-14 | 5.10E-11 |
| RELL1 | 379.43 | 0.66 | 0.09 | 7.58 | 3.47E-14 | 5.64E-11 |
| LINC01320 | 30.29 | 4.23 | 0.56 | 7.54 | 4.64E-14 | 7.19E-11 |
| DRP2 | 14.23 | -2.02 | 0.27 | -7.50 | 6.25E-14 | 9.25E-11 |
| MMP10 | 1644.52 | 3.55 | 0.48 | 7.41 | 1.29E-13 | 1.83E-10 |
| CCL26 | 204.12 | 5.28 | 0.71 | 7.38 | 1.57E-13 | 2.14E-10 |
| SOCS3 | 1019.16 | 3.21 | 0.44 | 7.36 | 1.80E-13 | 2.35E-10 |
| LYVE1 | 335.02 | 3.46 | 0.47 | 7.29 | 3.10E-13 | 3.91E-10 |
| ANKRD34A | 47.40 | -0.71 | 0.10 | -7.24 | 4.37E-13 | 5.32E-10 |
| PP7080 | 724.69 | 1.97 | 0.27 | 7.22 | 5.35E-13 | 6.28E-10 |
| PTPRG-AS1 | 45.12 | -1.29 | 0.18 | -7.20 | 6.25E-13 | 7.09E-10 |
| TTC12 | 815.43 | 0.79 | 0.11 | 7.10 | 1.27E-12 | 1.35E-09 |
| AL136040.1 | 42.18 | -1.26 | 0.18 | -7.10 | 1.27E-12 | 1.35E-09 |
| IGFL2 | 13.43 | -2.03 | 0.29 | -7.05 | 1.73E-12 | 1.78E-09 |
| ADGRE1 | 93.01 | 3.41 | 0.49 | 7.02 | 2.19E-12 | 2.19E-09 |
| SDK1 | 704.78 | 2.18 | 0.31 | 6.98 | 2.87E-12 | 2.65E-09 |
| ADAM28 | 6212.94 | 1.20 | 0.17 | 6.98 | 2.86E-12 | 2.65E-09 |
| HIPK3 | 5442.16 | 0.38 | 0.05 | 6.99 | 2.74E-12 | 2.65E-09 |
| SYNE2 | 16460.82 | 0.42 | 0.06 | 6.96 | 3.29E-12 | 2.95E-09 |
| FUZ | 181.23 | 1.43 | 0.21 | 6.95 | 3.70E-12 | 3.23E-09 |
| AC090643.2 | 11.87 | -2.76 | 0.40 | -6.93 | 4.28E-12 | 3.64E-09 |

BaseMean = mean RNA count, lfcSE = log2 Fold Change Standard Error, stat = Wald statistic Z-score, padj = p-value adjusted
